# A Novel Zirconia-Based Composite Presents an Aging Resistant Material for Narrow-Diameter Ceramic Implants

**DOI:** 10.3390/ma14092151

**Published:** 2021-04-23

**Authors:** Felix Burkhardt, Markus Harlass, Erik Adolfsson, Kirstin Vach, Benedikt Christopher Spies, Ralf-Joachim Kohal

**Affiliations:** 1 Medical Center—University of Freiburg, Center for Dental Medicine, Department of Prosthetic Dentistry, Faculty of Medicine, University of Freiburg, Hugstetter Str. 55, 79106 Freiburg, Germany; felix.burkhardt@uniklinik-freiburg.de (F.B.); markus.harlass@uniklinik-freiburg.de (M.H.); benedikt.spies@uniklinik-freiburg.de (B.C.S.); 2 Swerea IVF AB, Argongatan 30, 431 53 Mölndal, Sweden; erik.adolfsson@ri.se; 3 Medical Center—University of Freiburg, Institute for Medical Biometry and Statistics, Faculty of Medicine, University of Freiburg, Stefan-Meier-Str. 26, 79104 Freiburg, Germany; kv@imbi.uni-freiburg.de

**Keywords:** zirconia, ceramic implants, ceria-stabilized, loading/aging, scanning electron microscopy, fracture resistance

## Abstract

A novel ceria-stabilized zirconia-alumina-aluminate composite (Ce-TZP-comp) that is not prone to aging presents a potential alternative to yttrium-stabilized zirconia for ceramic oral implants. The objective of this study was to evaluate the long-term stability of a one-piece narrow-diameter implant made of Ce-TZP-comp. Implant prototypes with a narrow (3.4 mm) and regular (4.0 mm) diameter were embedded according to ISO 14801, and subgroups (n = 8) were subsequently exposed to dynamic loading (10^7^ cycles, 98N) and/or hydrothermal treatment (aging, 85 °C). Loading/aging was only applied as a combined protocol for the 4.0 mm diameter implants. One subgroup of each diameter remained untreated. One sample was cross-sectioned from each subgroup and evaluated with a scanning electron microscope for phase-transformation of the lattice. Finally, the remaining samples were loaded to fracture. A multivariate linear regression model was applied for statistical analyses (significance at *p* < 0.05). All samples withstood the different loading/aging protocols and no transformation propagation was observed. The narrow diameter implants showed the lowest fracture load after combined loading/aging (628 ± 56 N; *p* < 0.01), whereas all other subgroups exhibited no significantly reduced fracture resistance (between 762 ± 62 and 806 ± 73 N; *p* > 0.05). Therefore, fracture load values of Ce-TZP-comp implants suggest a reliable intraoral clinical application in the anterior jaw regions.

## 1. Introduction

At the same time when the first intra-osseous oral titanium implants were used to replace missing or deteriorated teeth [1], ceramic implants made of aluminum oxide (Al_2_O_3_) were developed [2,3]. However, compared to the titanium implants, these implants played only a subordinate role and did not prevail due to an increased risk of fracture and lack of osseointegration [4]. The demand for metal-free and aesthetically pleasing alternatives to titanium continued, and brought zirconia (ZrO_2_, zirconia) into focus as an oral implant material [5]. ZrO_2_ exhibits a crystalline lattice structure dependent upon environmental temperature conditions due to its allotropic properties [6]. With the addition of yttrium oxide (Y-TZP: yttrium stabilized tetragonal zirconia polycrystal), the tetragonal crystalline phase can be stabilized at room temperature. This allows the metastable ZrO_2_ grains to resist crack propagation (stress-induced phase transformation toughening, PTT) [7]. Therefore, Y-TZP exhibits exceptionally high strength (>1200 MPa) and can also be applied as an oral implant material [8,9,10]. However, in the warm and humid environment of the oral cavity, Y-TZP can undergo low temperature degradation (LTD), often referred to as aging [11]. This describes a spontaneous and continuously progressive transformation of ZrO_2_ grains from the tetragonal to the monoclinic phase in the presence of water molecules (*t-m* transformation) [12]. As a result, roughness and micro-cracks can occur on the surface. Consequently moisture can penetrate deeper layer-by-layer into the material and accelerate the aging phenomenon [13]. This may lead to a reduced fracture resistance of ceramic implants made of Y-TZP [14].

In order to avoid LTD in dental ceramics, a novel ceria-stabilized zirconia-based composite (Ce-TZP-comp) was developed within the European project titled *Longlife* (“Advanced multifunctional zirconia ceramics for long-lasting implants”, 7th European Framework Program) [15]. The development of the material was based upon the known positive properties of ceria-stabilized zirconia [16,17,18,19]. As a result of stabilization with cerium instead of yttrium, this novel zirconia-based material was not prone to aging [20]. The addition of two secondary phases, equi-axial alumina (α-Al_2_O_3_), and elongated strontium hexa-aluminate (SrAl_12_O_19_), to the ceria-stabilized zirconia matrix compensated for the reduced flexural strength [21]. An innovative powder coating process was developed for the fabrication of this material to create the ultra-fine composite structures [15]. To this end, zirconia powders were coated with precursors of the second phases, which crystallized on the surface of the zirconia particles under special thermal conditions. The compounds mentioned previously in combination with the refinement of the microstructure led to superior mechanical properties in terms of overall strength (>1 GPa) and toughness (>10 MPa√m) [21]. Furthermore, this zirconia-based composite exhibited an exceptionally high Weibull modulus (m = 60), which was typically characterized for metals at this scale [21]. Consequently, these mechanical properties indicate that the novel zirconia-based composite can be implemented clinically as an oral implant material. Therefore, the novel biomaterial was extensively evaluated with respect to its interaction with human and tissue-specific cells and oral microorganisms in vitro [20,22], which likewise suggested clinical applicability as an implant material. This was further substantiated by in vivo tests observing bone-to-implant contact and biomechanical implant stability after insertion into the femora of rats [20] and a fast osseointegration in the humeri of sheep [23]. Finally, a preclinical long-term evaluation of this novel biomaterial by loading implant-shaped specimens is necessary to guarantee clinical safety from a mechanical point of view prior to clinical application.

Since the insertion of regular diameter implants can be challenging in areas with limited bone volume, e.g., in the anterior region, narrow diameter implants may be indicated [24,25]. By using implants with a reduced diameter, the risk of bone dehiscence and fenestration can be reduced and bone augmentation might be avoided. Therefore, narrow diameter implants present an alternative in the anterior region as they showed comparable results in terms of survival and complication rates, as well as marginal bone loss compared to regular diameter implants [26,27]. However, data on the mechanical reliability of narrow diameter implants made of zirconia are sparse [28,29,30]. Therefore, the objective of the present investigation was to evaluate regular and reduced diameter implants made of this novel zirconia-based composite, particularly as it relates to its fracture resistance and potential changes after dynamical loading and hydrothermal aging. The postulated null hypotheses were that (i) the different loading/aging treatments in the chewing simulator have no effect on the fracture resistance and that (ii) no transformation zone can be observed at the surface of the evaluated implants.

## 2. Materials and Methods

### 2.1. Experimental Setup

**Figure 1:**
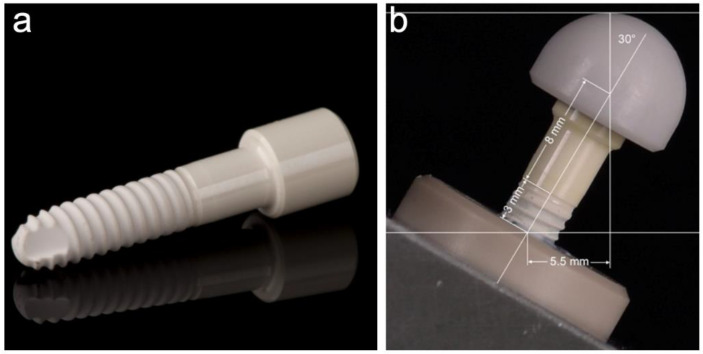
(**a**) Evaluated implant made of a ceria-stabilized zirconia-alumina-aluminate composite (Ce-TZP-comp), (**b**) embedding of the implants according to ISO 14801.

**Figure 2:**
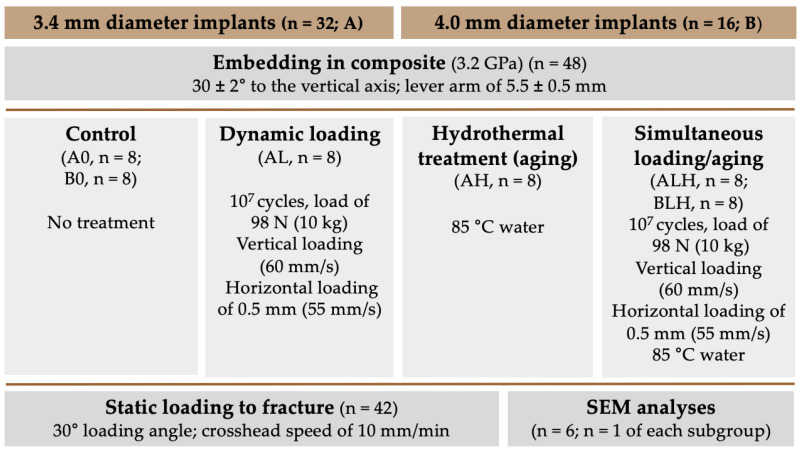
Schematic diagram of the experimental setup.

A total of 48 implant-shaped specimens made from a ceria-stabilized zirconia-alumina-aluminate composite (Ce-TZP-comp) with a diameter of 3.4 mm (A; n = 32) and 4.0 mm (B; n = 16) were used during the present investigation (Figure 1a). Group A was divided into four subgroups (n = 8 each), which differed from each other in the treatment of the implants. One subgroup was only dynamically loaded in a chewing simulator device (AL), while another subgroup was only hydrothermally treated (AH) in a water bath at 85 °C. Another subgroup received a combined treatment of dynamic loading and hydrothermal aging (ALH), whereas Group A0 remained untreated and served as the control group. Group B was divided into two subgroups (n = 8 each): one was hydrothermally treated and dynamically loaded (BLH), while the other subgroup was used as a control without treatment (B0). Subsequently, one implant from each subgroup was cross-sectioned and examined by scanning electron microscopy (SEM) (n = 6). With the exception of the samples used for cross-sections, all of the implants (n = 42) were loaded to fracture in a static loading test and statistically evaluated. Finally, fracture patterns were analyzed (n = 42). A schematic diagram of the experimental setup is shown in Figure 2.

### 2.2. Investigated Implants

The investigated implant prototypes were made of Ce-TZP-comp (kindly provided by the partners of the EU-SISCERA project) which contained equi-axial α-Al_2_O_3_ (22 vol.%) and elongated SrAl_12_O_19_ (8 vol.%) phases in a ceria-stabilized zirconia matrix. The synthesis of this novel Ce-TZP-comp was described in a prior publication [15]. Ce-TZP composite blanks were sintered at 1450 °C for 1 h at a heating and cooling rate of 5 °C/min. Finally, the implant prototypes were wet-grinded out of fully sintered Ce-TZP-comp composite blanks.

**Figure 3:**
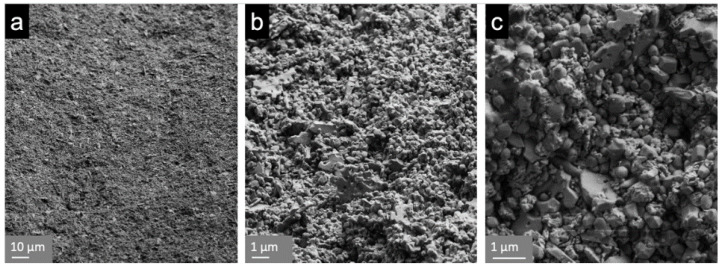
Scanning electron images (Supra 55VP, Zeiss, Oberkochen, Germany) of the blasted and etched endosseous surface of the implant prototypes with magnification of (**a**) ×500; (**b**) ×5000; (**c**) ×10,000.

The evaluated prototype (Figure 1a) consisted of a conical endosseous part (10.2 mm) that merged into a transgingival part (height: 5.0 mm) with a diameter of 3.4 mm (A) and 4.0 mm (B). The cylindrical part (5.0 mm length and 5.0 mm diameter) adjoining the transgingival part in this one-piece implant represents the abutment part. The endosseous part was blasted with Al_2_O_3_ (150 µm; 3.5 bar) and then immersed in an acid solution (7% hydrofluoric acid; 43% nitric acid) for 3 h. This resulted in a roughness (Ra) of approximately 1 μm, whereby the surface of the transgingival and abutment part was polished (Figure 3).

### 2.3. Embedding of the Samples

The 48 samples were embedded according to the ISO guideline 14801 [31], which was already described in detail in a previous publication [32]. The specimens were embedded with an angulation of 30 ± 2° to the vertical axis. The distance between the embedding plane and the loading center was 11 ± 0.5 mm and the lever arm measured 5.5 ± 0.5 mm (Figure 1b). In accordance with the ISO standard, a dual-curing composite (LuxaCore Automix Dual, DMG, Hamburg, Germany) with a modulus of elasticity above 3 GPa was used for the embedding in order to simulate the mechanical properties of human bone [33]. In addition, a recession of 3.0 mm was simulated by embedding the implants in such a way that 3.0 mm of the endosseous part was exposed above the composite level.

### 2.4. Dynamic Loading/Hydrothermal Treatment (-Aging)

The specimens of the subgroups AL, ALH and BLH were subjected to 10 million loading cycles with a load of 98 N (10 kg) in a chewing simulation device (CS-4.8, SD-Mechatronik, Feldkirchen-Westerham, Germany). Both horizontal and vertical forces were exerted to a loading hemisphere, which was attached to the cylindrical abutment part. One cycle consisted of a vertical loading of the hemisphere at its highest point (60 mm/s), followed by a horizontal movement of 0.5 mm at 55 mm/s. The antagonist had a plane surface and consisted of stainless steel. Thus, the contact at the highest tip of the loading hemisphere was realized even during horizontal movement. To achieve the loading cycles mentioned above, a loading process at a cycle frequency of 2 Hz for 58 days was required in the chewing simulator. Samples were inspected twice a day during the complete loading period. The test chambers of the simulation device (in which the loading of the samples was performed) were able to be heated and could be filled with water. Thus, the samples of the subgroups ALH and BLH were subjected to a simultaneous hydrothermal treatment in 85 °C water during the dynamic loading procedure; group AL was only dynamically loaded and not hydrothermally treated. The samples of group AH, however, were only treated in 85 °C water and were not subjected to dynamic loading.

### 2.5. Cross-Sectioning, Scanning Electron Microscopy (SEM)

To assess the composition of the implants with a scanning electron microscope (SEM), one implant from each subgroup was embedded in epoxy resin (EpoFix, Struers, Ballerup, Denmark). It was subsequently bisected on a precision cutting saw, using a 0.5 mm bronze-bonded diamond saw blade. The cut specimens were then ground and polished with diamonds and finished with a colloidal silica suspension (Struers Rotopol-22 equipped with Struers Rotoforce-4, Struers, Copenhagen, Denmark). Finally, the specimens were coated with carbon and examined with a field emission scanning electron microscope (Jeol JSM-7800F, Tokyo, Japan). The average size of the grains was measured with an image analysis software (Adobe Photoshop CS6, Adobe Inc., San José, CA, USA) from the SEM images. Approximately 150 randomly selected grains from each phase were measured. The average grain sizes of ZrO_2_ and α-Al_2_O_3_ were determined by applying the linear intercept method, using 1.56 as correction factor [34] according to the measurements of a precedent publication [21]. The average length and thickness of SrAl_12_O_19_ grains was recorded directly from the images without any correction.

### 2.6. Static Loading Test

With the exception of the specimens used for SEM analysis, all of the samples were loaded to fracture in a universal testing machine (Zwick, Z010/TN2S, Ulm, Germany). The specimens were loaded compressively with the same angle of 30° at a crosshead speed of 10 mm/min until failure.

### 2.7. Fracture Analyses

Fractured specimens were digitized with a 3D-profilometer (VR-500, Keyence, Osaka, Japan) and their fracture patterns were evaluated. As it relates to the direction of force application, the difference between the highest and lowest points of the step formation was measured.

### 2.8. Statistical Analyses

A one-way ANOVA was used to compare the different groups regarding “fracture load” and “bending moment”. For subsequent pairwise comparisons, the method of Bonferroni was applied in order to adjust for multiple testing. The calculations were conducted using the statistical software STATA 14.2 (StataCorp LP, College Station, TX, USA). *p*-values with *p* < 0.05 were set as statistically significant.

## 3. Results

### 3.1. Dynamic Loading Test

All of the tested implants survived the dynamic loading procedure with 10^7^ loading cycles and partly simultaneous hydrothermal aging.

### 3.2. Scanning Electron Microscopy (SEM)

**Figure 4:**
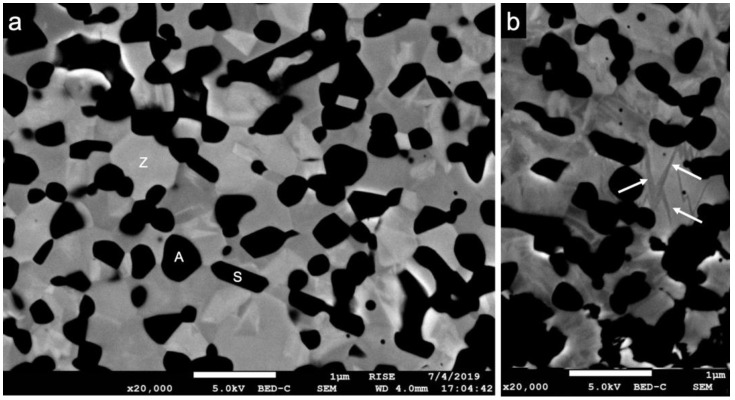
(**a**) SEM image (×20,000) showing the bulk material of the novel zirconia-based composite with pure alumina (A, dark equi-axial grains), strontium hexa-aluminate (S, dark elongated grains) and zirconia stabilized with cerium (Z, brighter grains). (**b**) SEM image (×20,000) displaying darker transformation bands in the zirconia grains after hydrothermal/mechanical treatment (white arrows).

**Figure 5:**
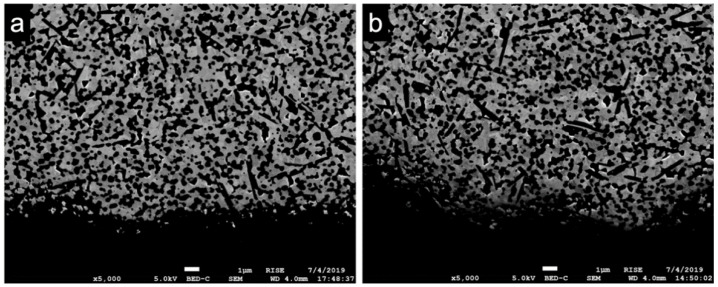
(**a**) SEM image (×5000) depicting the endosseous implant surface without treatment (group A0) and (**b**) after combined dynamical loading and hydrothermal aging (group ALH). No distinct transformation zone was observed after treatment.

SEM images revealed the expected three main phases of the novel zirconia-based composite material (Figure 4a). In addition to dark equiaxial grains (pure α-Al_2_O_3_), dark elongated grains (SrAl_12_O_19_) were observed. The brighter grains can be identified as pure ZrO_2_ stabilized with cerium, which was only found inside the grains and therefore was not visible in the images [15]. The average grain size of the pure α-Al_2_O_3_ grains was 0.5 ± 0.1 µm; average grain size of the Ce-stabilized ZrO_2_ was 0.8 ± 0.2 µm. The SrAl_12_O_19_ grains had a mean length of 1.4 ± 0.4 µm and a width of 0.3 ± 0.1 µm. No changes in grain size occurred because of the different mechanical and thermal treatments. In terms of the implant surfaces, no measurable *t-m* transformation occurred (Figure 5). However, in the bulk material some transformation bands were visible in the zirconia grains after loading and aging (Figure 4b). Irrespective of the mechanical or hydrothermal treatment, some specimens showed a slightly damaged surface at areas of the implant thread. This could have been caused by the blasting and etching treatment during the manufacturing process.

### 3.3. Static Loading Test

**Figure 6:**
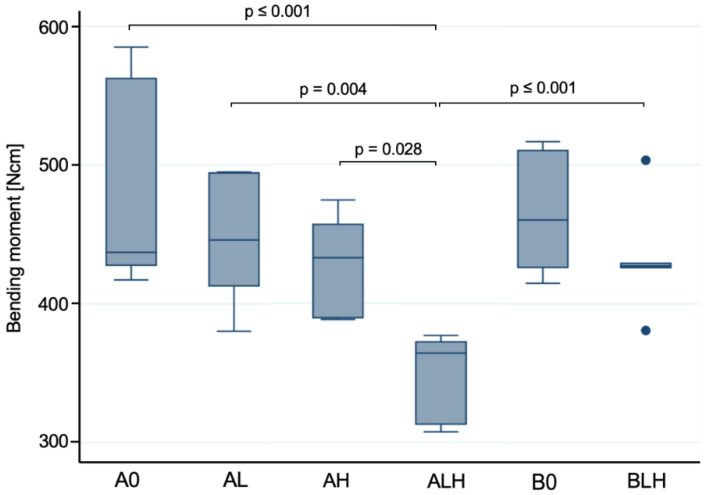
Boxplots showing the calculated bending moment of the static loading test (n = 7 per group; see Table 1 for detailed data). A whisker is drawn to display all samples lying within 1.5 times of the interquartile range, all other samples are shown as outliers. A one-way ANOVA was applied for statistical analyses and *p*-values of *p* < 0.05 were considered statistically significant.

Fracture load values and resulting bending moments are displayed in Figure 6 and Table 1.

In comparison to the untreated control samples A0, the simultaneously loaded and aged samples with 3.4 mm diameter (ALH) showed a significantly decreased fracture resistance (*p* < 0.001) in the static loading test. The solely dynamically loaded (AL) and solely hydrothermally treated (AH) samples within this group revealed no significant changes (*p* > 0.05) in fracture resistance with respect to each other, nor with respect to the control group (A0). Statistically significant differences were observed when comparing ALH with AH (*p* = 0.028) and AL (*p* = 0.004) regarding the fracture resistance. When comparing with group B, which differs in implant diameter, no significant differences were found between untreated samples A0 and B0 (*p* = 0.732). Likewise, no significant difference in fracture resistance following dynamic loading and hydrothermal aging (BLH) was observed compared to control B0 (*p* = 0.122). When comparing the two simultaneously loaded and aged groups BLH and ALH, the latter exhibited a statistically significant reduction in fracture resistance (*p* < 0.001).

### 3.4. Fracture Analysis

**Figure 7:**
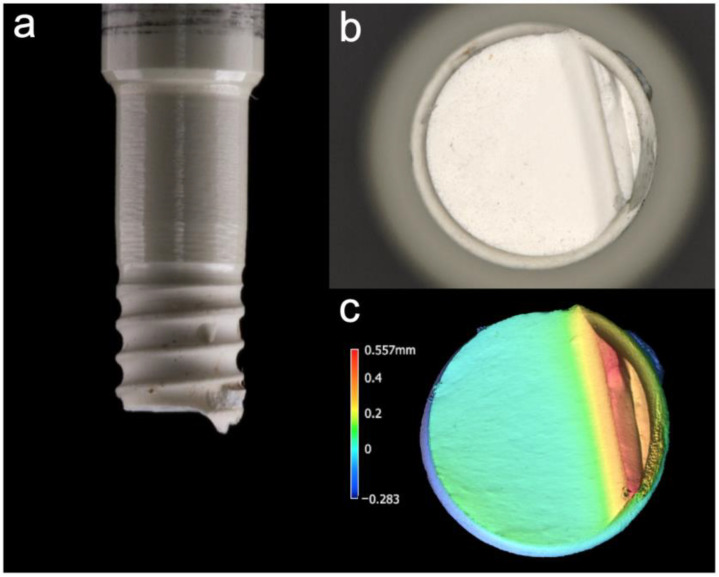
(**a**) Exemplary fracture evaluation of the Ce-TZP-comp implants: Side view of a smooth horizontal fracture in combination with a compression curl facing apically, (**b**) apical view of the fracture area (40× magnification), (**c**) profile analysis after digitization with a 3D profilometer (VR-500, Keyence, Osaka, Japan).

After the static loading test, all of the implants exhibited a similar fracture pattern. They fractured 1–2 mm below the embedding material (Figure 7). The fracture lines started from a thread valley located on the loading side and a smooth cut with a compression curl on the opposing side was observed. This indicates a fracture without major deviation of plane of cracking before the advancing crack enters the compression zone and curls off. The distance between the highest and lowest point of the fracture pattern was 0.43 ± 0.20 mm (A0), 0.50 ± 0.34 mm (AL), 0.72 ± 0.24 mm (AH), 0.45 ± 0.24 mm (ALH), 0.47 ± 0.13 mm (B0) and 0.66 ± 0.19 mm (BLH).

## 4. Discussion

The objective of the present study was to evaluate the long-term reliability of an implant prototype made from a novel zirconia-based composite in terms of (1) potential morphological changes observed in SEM and (2) fracture load/bending moment values.

### 4.1. Dynamic Loading and Hydrothermal Aging

Two ISO standards can be applied in this preclinical evaluation. ISO 13356 [35] describes the testing of simplified test specimens at 134 °C in a humid environment, whereby more complex geometries and surface post-processing are not taken into account. The second relevant ISO guideline, ISO 14801 [31], addresses a dynamic loading protocol, although the environmental conditions of the oral cavity are not considered. To evaluate the novel material as closely as possible in terms of its degradation in-vivo but in an accelerated manner, a previously described protocol [36] based upon the ISO standards mentioned above with combined loading and aging was applied. However, since the focus of this study was on the reliability of reduced diameter implants, they were additionally subjected to solely loading and solely aging to evaluate the effects on fracture strength.

The embedding of the implants was performed according to ISO standard 14801. Contrary to the ISO guideline, the chewing simulator applied horizontal forces in addition to vertical forces. To this end, the antagonist moved 0.5 mm horizontally in the opposite direction of the inclination angle of the implant during the application of the vertical load. This enabled a more realistic representation of the physiological chewing load [37]. The applied load of 98 N is within the range of mastication forces measured in vivo [38] and is consistent with comparable preclinical studies [36,39]. The influence of the warm and humid environment was investigated on the implants, given that this is known to cause aging in Y-TZP. For this purpose, instead of accelerated aging for 5 h at 134 °C as described in ISO 13356, a lower temperature of 85 °C over 58 days was chosen to more closely approximate in-vivo conditions. The fact that no fracture occurred in any of the implants after the various loading and aging protocols indicates a clinical reliability of the novel implant material for 10–40 years. This varies depending on the assumed number of loading contacts per year. The scientific data on this are very heterogeneous and range from 240,000 to 1 million per year [40,41,42].

### 4.2. Scanning Electron Microscopy

All of the examined samples showed an exceptionally fine-grained and homogeneous matrix revealing the three expected main phases (ceria-stabilized ZrO_2_, α-Al_2_O_3_, SrAl_12_O_19_). Cerium was only present in the ZrO_2_ grains, which was previously shown by Transmission Electron Microscopy and Energy-Dispersive X-ray Spectroscopy [15]. Pure Ce-TZP has a low strength due to larger grains and is thus unsuitable in loaded applications. Through the addition of the alumina and the aluminates, the grain growth of the zirconia grains during the densification could be inhibited. On the one hand, this fine-grained matrix composition can increase hardness and strength, while, on the other hand, the ability of phase transformation toughening is reduced. Thus, a precise amount of cerium is essential. At a cerium content of 10.5 mol%, the Ce-TZP-comp exhibited the best aging resistance and the ideal combination of strength and toughness, both of which are crucial factors in the fabrication of an oral implant material [21].

Following submission to the different loading and aging protocols, no distinct transformation zone with a transition of the zirconia grains from tetragonal to monoclinic was observed. This finding (absence of phase transformation zone) is consistent with previous observations that Ce-TZP/Al_2_O_3_-based nanocomposites showed no phase transformation when compared to Y-TZP after hydrothermal treatment in 121 °C vapor for 18 h [14]. In this regard, the α-Al_2_O_3_ grains may have contributed to the enhancement of the critical stress for phase transformation of Ce-TZP by “pinning” to the ZrO_2_ grains. Further, the SrAl_12_O_19_ grains may have counteracted crack initiation [15].

### 4.3. Static Load to Fracture

All of the examined implant prototypes with a diameter of 3.4 mm showed mean bending moments of ≥349 ± 29 Ncm whereas the 4.0 mm diameter implants revealed mean bending moments of ≥432 ± 36 Ncm. The deviation of the measured fracture load and calculated torque values was within a comparable range in all of the groups. Determining clinically acceptable loading values for oral implants is a highly discussed issue. In the anterior region, where primarily narrow diameter implants would be inserted, bite forces of 140 to 170 N were calculated for a single tooth [43,44]. Relative to the chewing forces measured in vivo [37], the obtained fracture load values of the Ce-TZP-comp implants were considerably higher in vitro (≥628 ± 56 N). The new implant prototype also showed promising results when compared to the mean bending moments from a current systematic review and meta-analysis for zirconia implants investigated in vitro [30]. Thus, a mean bending moment of 441.3 ± 152.7 Ncm was calculated for one-piece implants with a diameter of 3.8–4.4 mm, whereas implants up to 3.3 mm diameter had only 215.0 ± 6.7 Ncm. However, this data does not reflect whether the implants tested were thermally treated or dynamically loaded. In a laboratory study conducted according to ISO 14801, in which two-piece zirconia implants with reduced diameter (3.3 mm) were examined, fracture load values of 384.4 ± 52.8 N were measured [28]. In comparison, the one-piece Ce-TZP-comp implant with 3.4 mm showed high fracture load values (≥628 ± 56 N) even after simultaneous loading and aging, whereby the implant design has a decisive impact. In the present study, loading or hydrothermal aging of the narrow diameter implants (3.4 mm) resulted in slightly reduced fracture load values compared to the untreated implants. This difference, however, did not reach statistical significance. Only combined simultaneous loading and hydrothermal aging led to a significantly reduced fracture resistance of the narrow diameter implant compared to the respective control group and the implants subjected to separate loading and aging. It can therefore be assumed that, despite acceptable fracture load values, more severe fatigue can be induced by simultaneous loading and aging [36]. A critical stress level for the phase transformation of the zirconia grains might then be reached earlier and the ability for transformation toughening might be reduced during the final mechanical testing. Some transformation bands located in the zirconia grains of the bulk material indicate a presence of the monoclinic phase. This suggests an inhomogeneous distribution of the monoclinic phase to a certain extent, which does not appear as a transformation layer at the surface as it is usually the case for Y-TZP ceramics [45,46]. However, again the implant diameter seems to have a decisive impact as well, because the 4.0 mm diameter prototypes did not exhibit a significantly reduced bending moment following the same simultaneous loading and aging. This might be explained by higher stress levels in the narrow diameter implants compared to the 4.0 mm diameter implants since the same load was used in the final loading test. The critical stress for the transformation might not have been reached in the 4.0 mm implants, resulting in significantly higher bending moments.

The one-piece design could have been another decisive factor contributing to these high fracture load values, as a higher fracture resistance was observed for one-piece implants in most cases [30]. This might have been due to the absence of an implant-abutment connection as a possible weak point and the use of just one material, which prevented different aging behavior. However, the one-piece design has confined indications due to the non-submerged healing and the limited correction of the abutment axis after insertion [47]. Today, extensive research is being conducted to determine the precise type of surface topography and modifications that yield the best soft tissue integration and osseointegration of Ce-TZP implants [22,48,49]. In this context, further studies are needed to evaluate whether surface modifications such as different roughness parameters have an influence on the biomechanical stability of Ce-TZP oral implants.

Taken together, with respect to the above-mentioned hypotheses, the first aspect was rejected by our findings, since narrow diameter implants showed a reduced fracture resistance after simultaneous loading and aging. The second aspect can be considered as verified since no transformation zone was observed at the implant surface. The novel zirconia composite material therefore showed overall very reliable mechanical data for narrow diameter implants and thus represents an aging-resistant alternative to market-available zirconia implants.

## 5. Conclusions

Within the limitations of the present study, the novel ceria-stabilized zirconia implant prototypes evaluated showed no distinct transformation zone after separate and combined loading and aging. This was also reflected in the fracture load values, which were not significantly reduced with the exception of the narrow diameter implant after simultaneous loading/aging. However, from a mechanical point of view, fracture load values of 628 N for the narrow implant indicate a reliable intraoral clinical application. In addition to the novel fine-grained zirconia composite, the one-piece design may have contributed to the promising long-term stability.

## Figures and Tables

**Table 1:** Average values and standard deviations of load and calculated bending at the time of fracture in the static loading test for the evaluated groups. Values labeled with the same superscript letter indicate no significance (significance at *p* ≥ 0.05).

Groups	A0	AL	AH	ALH	B0	BLH
Load (N)	854 ± 116 ^a^	806 ± 73 ^a^	762 ± 62 ^a^	628 ± 56 ^b^	845 ± 70 ^a,c^	782 ± 60 ^c^
Bending Moment (Ncm)	477 ± 70 ^a^	448 ± 44 ^a^	427 ± 35 ^a^	349 ± 29 ^b^	466 ± 40 ^a,c^	432 ± 36 ^c^

## Data Availability

The data presented in this study are available on request from the corresponding author.
